# Posterior percutaneous endoscopic versus traditional surgery for cervical disc herniation: A protocol for systematic review and meta analysis

**DOI:** 10.1097/MD.0000000000021442

**Published:** 2020-07-31

**Authors:** Feng-Qi Sun, Shao-Jin Wen, Bing-Lin Ye, Chen-Xu Li, You-Fu Fan, Yong-Sheng Luo, Xiang-Fu Wang

**Affiliations:** a Department of Spine Minimally Invasive Orthopedics; b Department of Hematology, Gansu provincial Hospital of Tradition Chinese Medicine, Lanzhou, China.

**Keywords:** meta-analysis, posterior percutaneous endoscopic, protocol, systematic review, traditional surgery

## Abstract

**Background:**

Posterior percutaneous endoscopic cervical diskectomy (P-PECD) can be used posterior microdiscectomy for cervical disc herniation. But only some small sample sizes of clinical studies have evaluated the efficacy and safety of P-PECD. This study aim to evaluated the efficacy and safety of P-PECD compared with traditional open surgery.

**Methods:**

We will search the following seven electronic databases from their initiation to the May 1, 2020: PubMed, Embase, Cochrane Library, Web of Science, China National Knowledge Infrastructure (CNKI), Chinese Biomedical Literature Database (CBM) and Wanfang database. All randomized controlled trials, non-randomized controlled trials and retrospective case controls that compared the efficacy and safety of P-PECD and traditional open surgery in the treatment of cervical disc herniation will be included. The pooled odds ratio with 95% credible intervals (CIs) was used for the dichotomous variables. The mean difference with 95% CIs was used for the continuous variables. All analyses were conducted by Comprehensive Meta Analysis 2.0. A 2-tailed *P* value < 0.05 is considered statistically significant.

**Results:**

The results of systematic review and meta-analysis will be submitted to a peer-reviewed journal.

**Conclusion:**

Our study will provide clarity regarding for clinicians to choices best surgical approach for patients with cervical disc herniation. Any changes that need to be made during the process of this study will be explained in the final full-text publication.

**Protocol registration number:**

CRD42020164011.

## 1 Introductions

In 1975, Hijikata first introduced percutaneous lumbar nucleotomy.^[1]^ Since then, percutaneous discectomy has been developed for the treatment of disc disease in the lumbar, thoracic spine and cervical.^[2–4]^ Compared to open microsurgery, endoscopic spine surgery is now considered an effective alternative surgery for the treatment of various lumbar disc herniations. Technological advances in recent decades have enabled the development of new technologies not only to achieve clinical outcomes similar to traditional surgery, but with the advantages of shorter hospital stay, reduced blood loss, earlier functional recovery and less tissue damage.^[5–7]^

Unlike used this technologies in lumbar disc herniations, cervical spine surgery has some unique complications. Such as dysphagia, unilateral recurrent laryngeal nerve palsies, accidental esophageal perforation, cerebrospinal fluid leakage and temporary unilateral Horner syndrome.^[8,9]^ However, posterior percutaneous endoscopic cervical diskectomy (P-PECD) may avoid the above complications. Especially the improvement of the endoscopic instruments has allowed the P-PECD increases its usefulness.^[10]^ Therefore, P-PECD can be used as an alternative to posterior microdiscectomy for cervical disc herniation. But, to our knowledge, only some small sample sizes of clinical studies have evaluated the efficacy and safety of P-PECD.^[11,12]^ The status is a big obstacle for clinicians to choose reasonable surgical methods.

Systematic reviews (SRs) and meta-analysis (MAs) is a new analysis ways that using systematic approaches to identify, select and critically appraise primary studies.^[13,14]^ Now, SRs and MAs are the basic tools for generating reliable medical information,^[15]^ which provides a synthesis of a large amount of evidence to help clinicians keep pace with the medical literature, explain the differences between studies on the same issue, formulate clinical policies, combine best evidence with clinical practice, and suggest directions for new study.^[16,17]^

Therefore, we designed this SRs and MAs to evaluated the efficacy and safety of P-PECD compared with traditional open surgery. We hope that the results of our study can provide a reference for clinicians.

## 2 Methods

The study will be conducted in line with the recommendation of Preferred Reporting Items for SRs and Meta Analyses guidelines.^[18]^ The methodology for constructing the study’ protocol followed the criteria established by the Preferred Reporting Items for SRs and Meta-Analyzes Protocols.^[19]^ This systematic review protocol was registered in the International Prospective Register of SRs (https://www.crd.york.ac.uk/PROSPERO/), under the protocol number: CRD42020164011.

### 2.1 Inclusion criteria

#### 2.1.1 Types of patients

We will include patients with cervical disc herniation that were diagnosed using any recognized diagnostic criteria. Patients also need to meet the following conditions:

(1) Age≥18 years;
(2) patients cannot with a lumbar surgery history, infection, tuberculosis, tumors, and other diseases.

In addition, no sex, race, or socioeconomic status restriction will be applied.

#### 2.1.2 Types of interventions

P-PECD defined as follows: Patients were placed in the prone position with appropriate flexion on a radiolucent surgery table. The surgeries were performed under local anaesthesia to allow for monitoring of any changes in the patients symptoms and signs during the procedure. A thin working sheath is completely inserted percutaneously through a stab incision. A working-channel endoscope is then placed in the working sheath. Surgical instruments are then introduced through the working channel. The surgical field is always visualized using a monitor system. The procedure is performed under continuous saline irrigation.^[20]^

In our MAs, only P-PECD will be included. PECD via the lateral approach or PECD combined with lumbar interbody fusion will be excluded.

#### 2.1.3 Types of controls

The control group had to be patients who had received traditional open surgery.

#### 2.1.4 Outcomes

Primary outcome is efficacy, including Back and Leg Visual Analog Scale (VAS) score,^[21]^ Japanese Orthopedic Association (JOA) score,^[22]^ the Oswestry Disability Index (ODI),^[23]^ and MacNab criteria.^[24]^

Secondary outcome is safety, which incidence of complications, including dura tear, incomplete decompression, reoperation, incidental durotomy, epidural hematoma, headache, infection, recurrence rate.

The patients of the included study were followed for at least 1 year.

#### 2.1.5 Types of studies

This study plan to include all randomized controlled trials (RCTs), non- RCTs and retrospective case controls that compared the effectiveness and safety of P-PECD and traditional surgery in the treatment of cervical disc herniation. Observational studies (eg, case series, case report) will be excluded. In addition, there studies that were not peer-reviewed or cannot retrieve relevant data (eg, letters, comments, and conference proceedings) will also be excluded. Finally, the study only published in English or Chinese will be eligible for inclusion.

### 2.2 Information sources and literature search

A comprehensive literature search will be carried out in the following seven electronic databases from their initiation to the May 1, 2020: PubMed, Embase, Cochrane Library, Web of Science, China National Knowledge Infrastructure (CNKI), Chinese Biomedical Literature Database (CBM) and Wanfang database. There are no restrictions on the publication date of the literature, including online literature in advance, and the publication language is limited to Chinese and English. The main search terms including “Cervical disc herniation”, “Cervical disc herniations”, “percutaneous spinal endoscopy” and “percutaneous endoscopic cervical discectomy”. Detailed search strategy of PubMed has been exerted in Table 1. We will modify similar search strategies for other electronic databases. In addition, to avoid missing potential trials, we will also retrieve conference papers, dissertations, ongoing studies, and reference list of all related reviews.

**Table 1:**
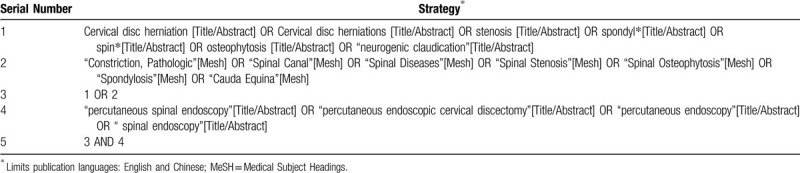
Sample search string for PubMed.

### 2.3 Study selection

**Figure 1:**
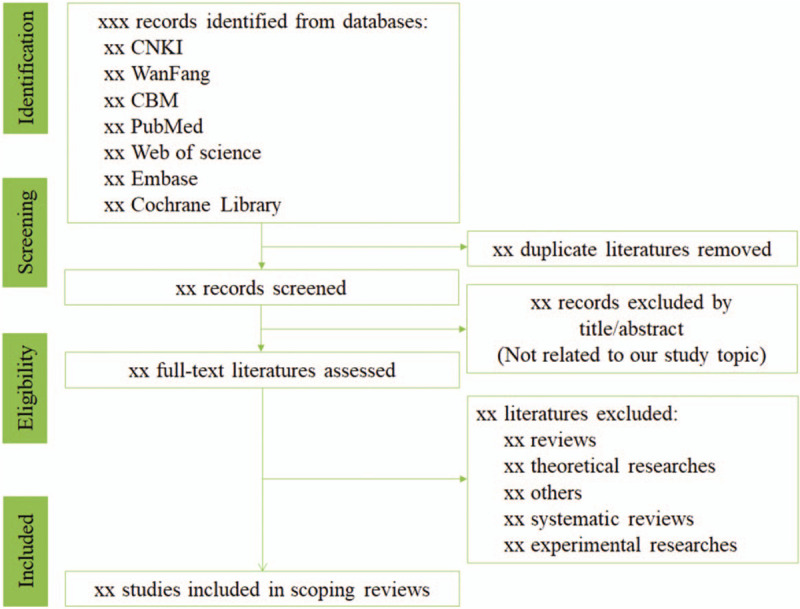
Flow diagram of study selection.

The study selecting process will be entire according to the flow of Figure 1. All the works of study selecting will be done independently by two reviewers. Any conflict will be resolved by discussion with the help of another reviewer. First, all collected records will be imported into EndNote X8 and all duplicated records will be removed. Second, records will be screening to rule out obvious nonconformities by titles and abstracts. Finally, we will obtain full-texts of remaining studies and carefully examine them according to the inclusion criteria.

### 2.4 Data extraction

The essential data will be extracted for all eligible trials. A predetermined sheet of data collection will be used to extract data independently by 2 reviewers. Any inconsistencies will be discussed and negotiated with another reviewer. The extracted data includes title, first author, publication year, sample size, patient characteristics (eg, race, sex, and age), interventions, therapeutic regimens, outcomes, and other relevant data.

### 2.5 Dealing with missing data

About the missing or incomplete data, we will contact original corresponding authors of included study to obtain it.

### 2.6 Data synthesis and analysis

We conducted meta-analyses using the Mantel–Haenszel method with the random-effects model for RCTs and case-control trials to estimate the overall effect size. The pooled odds ratio with 95% credible intervals (CIs) was used for the dichotomous variables. The mean difference with 95% CIs was used for the continuous variables. The heterogeneity between trials was evaluated using *I*^2^ statistics. The values of 25%, 50%, and 75% for the *I*^2^ as indicative of low, moderate, and high statistical heterogeneity, respectively.

We explored the publication bias using the Egger test and funnel plots if the number of included studies exceeded nine. All analyses were conducted by comprehensive meta analysis 2.0. A 2-tailed *P* value < 0.05 is considered statistically significant.

### 2.7 Subgroup analysis

If the necessary data are available, we would perform subgroup analysis and meta-regression analysis to assess whether the age of patients and publication language is the sources of heterogeneity or affect the results.

### 2.8 Study quality assessment

The Risk of Bias assessment tool from the Cochrane Handbook was used to assess the methodological quality of RCTs,^[25]^ and the Newcastle-Ottawa Scale was used to assess the quality of case controls.^[26]^ Each RCT was assessed to low risk, high risk, or unclear risk relating to the following items: sequence generation, allocation concealment, blinding of outcome assessors, incomplete outcome data, selective outcome reporting, and other sources of bias. The NOS assesses the quality of case controls with eight questions in 3 broad categories:

(1) patient selection;
(2) comparability of study groups;
(3) assessment of the outcome.

The total score is 9, the higher the score, the better the quality of the study. Two reviewers will independently to complete the quality assessment. Any disagreement between the reviewers will be resolved by discussion or consultation with another reviewer.

### 2.9 Quality of evidence

The grading of recommendations assessment, development, and evaluation will be used to assesse the quality of evidence for all outcomes.^[27]^ It mainly considerations including: risk of bias, inaccuracy, inconsistency, indirectness, publication bias. The quality of evidence will be graded 4 levels: very low, low, moderate, and high level.

### 2.10 Ethics and dissemination

Because this study is not a clinical study, and we will search and evaluate only existing sources of literature. So, ethical approval is not required.

## 3 Discussion

We anticipate the outcome of our study provide clarity regarding for clinicians to choices best surgical approach for patients with cervical disc herniation. This is also importance for laying foundation for further studies. This is just our study protocol and which is currently in piloting of the study selection process. Any changes that need to be made during the process of this study will be explained in the final full-text publication.

## Acknowledgment

The authors thank all investigators and supporters involved in this study.

## Author contributions

**Data curation:** Feng-Qi Sun, Xiang-Fu Wang

**Formal analysis:** Shao-Jin Wen

**Funding acquisition:** Xiang-Fu Wang

**Methodology:** Feng-Qi Sun, Bing-Lin Ye, Chen-Xu Li

**Resources:** You-Fu Fan, Yong-Sheng Luo

**Software:** Feng-Qi Sun, Shao-Jin Wen

**Writing – original draft:** Feng-Qi Sun

**Writing – review and editing:** Feng-Qi Sun, Xiang-Fu Wang
